# Patellofemoral contact patterns before and after total knee arthroplasty: an in vitro measurement

**DOI:** 10.1186/1475-925X-12-58

**Published:** 2013-06-26

**Authors:** Arnd Steinbrück, Christian Schröder, Matthias Woiczinski, Andreas Fottner, Peter E Müller, Volkmar Jansson

**Affiliations:** 1 Department of Orthopaedic Surgery, University Hospital of Munich (LMU), Campus Grosshadern, Marchioninistr. 15, Munich 81377, Germany

**Keywords:** Patella, Total knee arthroplasty, Patella tracking, Retropatellar pressure, Trochlear groove, Knee rig

## Abstract

**Background:**

Patellofemoral complications are one of the main problems after Total Knee Arthroplasty (TKA). Retropatellar pressure distribution after TKA can contribute to these symptoms. Therefore we evaluated retropatellar pressure distribution subdivided on the ridge, medial and lateral surface on non-resurfaced patella before and after TKA. Additionally, we analyzed axial femorotibial rotation and quadriceps load before and after TKA.

**Methods:**

Seven fresh frozen cadaver knees were tested in a force controlled knee rig before and after TKA (Aesculap, Tuttlingen, Germany, Columbus CR) while isokinetic flexing the knee from 20° to 120° under weight bearing. Ridge, medial and lateral retropatellar surface were defined and pressure distribution was dynamically measured while quadriceps muscles and hamstring forces were applied. Aside axial femorotibial rotation and quadriceps load was recorded.

**Results:**

There was a significant change of patella pressure distribution before and after TKA (p = 0.004). In physiological knees pressure distribution on medial and lateral retropatellar surface was similar. After TKA the ridge of the patella was especially in higher flexion grades strongly loaded (6.09 +/−1.31 MPa) compared to the natural knee (2.92 +/−1.15 MPa, p < 0.0001). Axial femorotibial rotation showed typical internal rotation with increasing flexion both before and after TKA, but postoperatively it was significantly lower. The average amount of axial rotation was 3.5° before and after TKA 1.3° (p = 0.001). Mean quadriceps loading after implantation of knee prosthesis did not change significantly (575 N ±60 N in natural knee and after TKA 607 N ±96 N; p = 0.28).

**Conclusions:**

The increased retropatellar pressure especially on the ridge may be one important reason for anterior knee pain after TKA. The trochlea of the femoral component might highly influence the pressure distribution of the non-resurfaced retropatellar surface. Additionally, lower axial femorotibial rotation after TKA might lead to patella maltracking. Changing the design of the prosthesis or a special way of patella shaping might increase the conformity of the patella to trochlea to maintain natural contact patterns.

## Abstract (de)

**Abstract:**

**Hintergrund**

Das patellofemorale Schmerzsyndrom stellt eines der zentralen Probleme in der Knieendoprothetik dar. Dabei wird angenommen, dass die retropatellare Druckverteilung nach Implantation einer Knietotalendoprothese (KTEP) zu dieser Symptomatik beiträgt. Wir untersuchten daher die retropatellare Druckverteilung unterteilt in First, mediale und laterale Rückfläche vor und nach KTEP. Zusätzlich analysierten wir die tibiofemorale Rotation und die Quadrizepszugkraft vor und nach KTEP.

**Material und methoden**

Die Simulation der Kniebewegung erfolgte an sieben humanen fresh-frozen Kniegelenken mit einem kraftgesteuerten Kniegelenkskinemator von 20° bis 120° Flexion mit programmierter Regelung unter konstanter isokinetischer Gewichtskraft an den Sehnen des M. quadriceps, M. semitendinosus und M. biceps femoris. Nach standardisierter Flächendefinition des Firstes, der medialen und lateralen Retropatellarfläche wurde die retropatellare Druckverteilung mittels Druckmessfolie vor und nach Implantation einer KTEP dynamisch gemessen. Zusätzlich wurden die tibiofemorale Rotation, sowie die Quadrizepszugkraft analysiert.

**Ergebnisse**

Nach Implantation einer KTEP zeigte sich eine signifikante Änderung der retropatellaren Druckverteilung. Im natürlichen Kniegelenk war der retropatellare Druck medial und lateral gleichmäßig verteilt. Postoperativ zeigte sich bei höheren Flexionsgraden ein maximaler retropatellarer Druck besonders im Bereich des Patellafirstes (6,09 +/−1,31 MPa) im Vergleich zum natürlichen Kniegelenk (2,92 +/−1,15 MPa, p < 0,0001). Die axiale tibiofemorale Rotation zeigte die typische Innenrotation bei Flexion und Außenrotation bei Extension sowohl vor als auch nach Implantation einer KTEP; postoperativ war diese Rotation jedoch deutlich vermindert. Die durchschnittliche axiale Rotation betrug 3,5° vor und 1,3° nach KTEP-Implantation (p = 0,001). Die Quadrizepszugkraft änderte sich nach KTEP-Implantation nicht signifikant (575 N ±60 N beim natürlichen Knie und nach KTEP 607 N (±96 N), p = 0,28).

**Schlussfolgerung**

Die Implantation einer KTEP führte in unserer Studie zu einer Steigerung des retropatellaren Drucks insbesondere im Firstbereich. Es ist davon auszugehen, dass durch die Erhöhung des retropatellaren Drucks und die ungünstigere Druckverteilung in vivo postoperative Patella Beschwerden entstehen können. Daneben kann die postoperativ verminderte Innenrotation der Tibia bei Flexion des Kniegelenkes zum Patella Maltracking führen. Wird bei der Implantation einer Knieprothese kein Retropatellarersatz durchgeführt ist ein standardisiertes knöchernes “Trimming” der Patella entsprechend dem Trochlea-Design der Prothese zu diskutieren.

## Background

Although Total Knee Arthroplasty (TKA) is a successful solution for osteoarthritis of the knee up to 19% of primary TKA patients are not satisfied with the outcome [1]. Patellofemoral complications after TKA are still a main cause of failure [2] and include chronic anterior knee pain, fracture of the patella, patella clunk syndrome, patella luxation and subluxation, as well as rupture of patella tendon [3-6]. Boyd et al. found 12% of peripatellar complications after TKA without resurfacing [3]. Revision rates and postoperative outcome, however, are similar for resurfaced and nonresurfaced patella [7,8]. Regarding anterior knee pain several authors emphasize that the postoperative outcome is rather influenced by femoral component design than by retropatellar resurfacing [7,9,10]. It is assumed that excessive postoperative retropatellar pressure contributes to a great extent to postoperative patella problems [9,11-13]. In vitro studies with cadaver knees are a successful method to analyze this pressure distribution [12,14], and were also used to investigate retropatellar contact patterns in this study.

Studies with fluoroscopy also showed that patellofemoral kinematics are modified after TKA [15]. In this context, Dennis et al. argue that femorotibial rotation is especially important for a normal patellar tracking and to prevent patellofemoral shear forces [16]. A decrease of femorotibial rotation after TKA might be one reason for patella maltracking and anterior knee pain.

Flexion and extension moment are also altering after TKA [17]. In particular, quadriceps strength is supposed to be an important factor for a well-functioning TKA and to prevent postoperative patella problems [18]. Ostermeier et al. showed in their study [19] an increase of 9% of quadriceps load for the posterior cruciate-retaining fixed-bearing TKA group.

To the authors knowledge there is no in vitro study which investigates retropatellar pressure distribution subdivided in ridge, medial and lateral surface, femorotibial rotation and quadriceps load before and after TKA with fresh-frozen specimens during a simulated squat of 100° of range of motion. Therefore, we focused on three aspects in our knee rig study: First we analyzed retropatellar contact patterns subdivided in ridge, medial and lateral surface in natural knees and after implantation of knee prostheses to evaluate this specific TKA design. Then we continued with investigating axial femorotibial rotation, which was assumed to alter after TKA. Finally, flexion and extension force of the quadriceps muscle was analyzed before and after TKA. The in vitro analysis was performed with fresh-frozen specimens dynamically in a special knee rig under force controlled muscle loading in a range of motion of 20° to 120° of knee flexion.

## Methods

Seven fresh frozen knee specimens (age range between 38 and 68 years, 3 female, 4 male) were resected about 20 cm proximal and 15 cm distal to the joint line, preserving articular capsule, ligaments and tendons surrounding the knee joint. Fibula head was fixed to tibia with a standard 4.5 diameter screw. Muscle- and fat-tissue was carefully removed from the tendons and bones. After on metallic finger traps (Bühler-Instrumente Medizintechnik GmbH, Tuttlingen, Germany) were connected to the tendons and suture material (FibreWire, Arthrex, Munich, Germany) was used to fix the tendon into the metallic mesh of the traps. The femoral and tibial bones were embedded into a metallic pot both fixed with epoxy casting resin (Rencast FC53, Huntsman, Basel, Switzerland).

**Figure 1:**
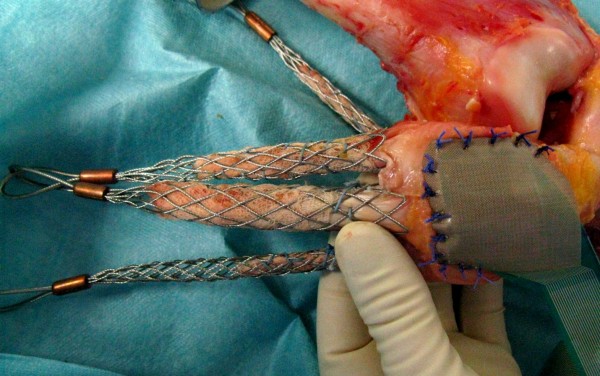
Photograph of a specimen after the finger traps were connected to the prepared tendons and the pressure sensitive film was attached to the retropatellar surface.

Shaping of the patella was performed by removing existing osteophytes on the patella circumference. The patella ridge was not shaped and patella remained unresurfaced. Afterwards a pressure sensitive film (K-Scan 4000, Tekscan Inc., Boston, USA) was attached to the retropatellar surface by subcutaneous 1.0 suture material. For stable suturing and to avoid shear forces a 0.1 mm Teflon tape (PTFE-tape) was glued on the sensor before (Figure 1). The sensor was conditioned and calibrated with a two point calibration (error of the root mean square 2.7% [20], nonlinearity max. 3%) as recommended by the manufacturer using a material testing machine (Z010, Zwick, Ulm, Germany). The maximum pressure of the sensor film was 1500 PSI (~10 MPa) with 572 total number of sensels (62 sensel per cm^2^).

For definition of the ridge of the patella first the highest point of the ridge of the patella was located with a pen also used for digital devices as smartphones. Afterwards the course of the ridge was followed from proximal to distal. This sweeping was recorded by the software of the sensitive film and repeated three times. Then the borders of the medial and lateral retropatellar surface were located on the retropatellar pressure sensor. From the middle of the course of the ridge (width: 1.3 mm = 1 sensel) we added 2.6 (2 sensels) mm medial and lateral to define the overall width of 7.5 mm (5 sensels) of the patella ridge and the borders to medial and lateral retropatellar surface to differentiate patellofemoral contact points.

**Figure 2:**
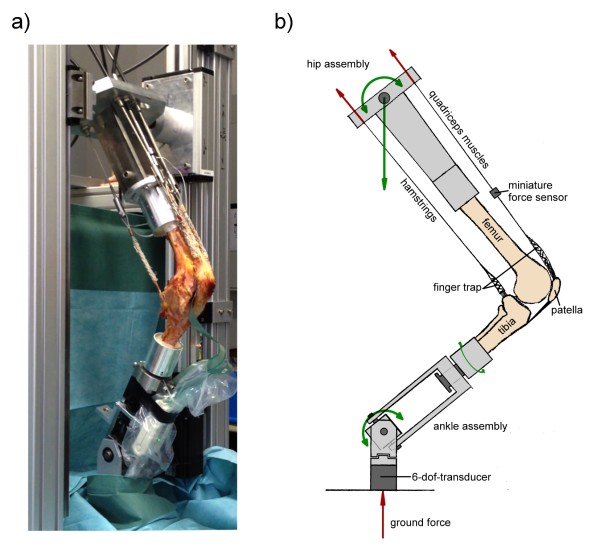
**Composition of the Munich knee rig a) natural specimen with a pressure sensitive film on the retropatellar surface (Tekscan Inc.) tested in the rig b) schematic drawing of the rig illustrating the simulated muscles in the sagittal plane.** Forces are shown in red and degrees of freedom are represented in green arrows. (Represented in the drawing are: flexion/extension of femur and tibia, internal/external rotation of the tibia, proximal/distal translation of the femur; not represented are: medial/lateral translation and varus/valgus position of the tibia).

The prepared knee was then mounted into the specially built knee simulator with 6 degrees of freedom (dof) (Figure 2a + b). This knee rig simulates a loaded squat using two linear drives (Driveset M150/180, Systec GmbH, Muenster, Germany; max. axial static load 2500/5000 N; precision of 0.1/0.1 mm; max. velocity 0.1/0.35 m/s). The first actor flexes and extends the knee with a constant velocity of 3°/s. Two angle sensors (8820 Burster, Gernsbach, Germany; capacity 350°, nonlinearity 0.5%, resolution 0.01°) in the simulated upper “hip-assembly” and simulated lower “ankle-assembly” were used to measure the flexion angle of the knee joint and axial femorotibial rotation. The second drive simulates the quadriceps muscle; the actual force was measured with miniature force transducers (8417–6002 Burster, Gernsbach, Germany; max. load 2000 N, nonlinearity 0.5%) near the tendons. Additionally, muscle forces of the hamstrings (semitendinosus and biceps femoris muscle), as well as lateral vastus and medial vastus muscle were simulated with 2 kg weights. A 6 degrees of freedom force-moment-sensor (FN 7325–31 FGP Sensors, Cedex, France) measured the ground reaction at the ankle assembly. This sensor has a capacity of 5000 N (axial), 2500 N(x/y plane), 65 Nm (torque/bending) with a nonlinearity of 1.0%. All sensors were amplified up to max 10 volts and digitalized with an 18–bit (262144 increments) analog digital converter using 8 differential analog input channels (PCI 6281 National Instruments, Texas, USA; min. accuracy: 28 μvolts). The rig was controlled with a personal computer with a self-programed code with LabVIEW 8.6 (National Instruments, Austin, Texas, USA). The knee was moving with a 50 ms PID- force- control loops to hold the ground reaction force constant to 50 N with the loaded quadriceps muscle. The data of the sensors were collected and written in an ASCII file.

Due to the results of Müller et al. [21] and Victor et al. [22,23] that kinematic profiles are similar with different ground forces for this study all specimens were tested with a ground reaction force of 50 N, which is comparable to the study of Yildirim et al. [24]. The squat from 20° to 120° flexion and back to 20° again was performed with a velocity of 3°/s.

After measuring retropatellar pressure in the natural knee during squatting, each specimen was removed of the rig and TKA was performed. We implanted a fixed bearing, cruciate-retaining TKA (Aesculap, Tuttlingen, Germany, Columbus CR) through a medial subvastus approach in tibia first technique. In coronal plane, the tibial resection was perpendicular to the bone axis in neutral rotation using an intramedullary rod. The femoral component was mounted with a 4°-6° valgus relative to the axis of femoral shaft using an intramedullary rod. All chosen specimen had no serious valgus or varus deformity (≤15°), the osteoarthritis was between 0° and 2° Kellgren & Lawrence Score [25]. The height range of the specimen-donators was between 170 and 177 cm, the weight range was between 62 and 93 kg. The local ethics committee of the University LMU München approved the acquisition and usage of the human specimens. Femoral component was implanted in a neutral rotation with no external rotation referenced to the transepicondylar axis. According to the knee size the authors implanted three times femur size 4 and three times size 5, once femur size 6. The femur size of the knee system ranges from 1 to 7. Tibial we implanted four times size 4, two times size 3, once tibia size 2. The tibia size ranges from 1 to 5. The knees were balanced using a gauge instrument. A 10 mm polyethylene inlay was used in every TKA. All knees were operated by the author A.S., who is in his 8th post-graduate year and a specialist for orthopedic and trauma surgery. All knees were operated by the author under assistance of author A.F, who is the senior attending of knee and hip arthroplasty in our department.

The evaluation after TKA was performed under the same conditions as the natural knee. All specimen were x-rayed anterior-posterior, sagittal and sunrise view by fluoroscopy before implantation of the prosthesis (to evaluate the grade of osteoarthritis and patellaalignment) and after TKA (to control the correct implantation of TKA).

### Statistical methods

All results were represented with the mean and standard deviation. The functional relation between flexion angle (FA) and the measured parameters were modeled by means of mixed effects models using a random intercept per specimen. Fixed effects included in the model were the cosine of the flexion angle, the squared cosine of the flexion angle, the cubed cosine of the flexion angle, and the presence of TKA (yes = 1/no = 0). Separate models were fitted for flexion (=0) and extension (=1). These analyses were performed using SAS version 9.2 for Linux (SAS Institute, Cary, NC). The regression coefficients to the statistical model including the p-values are summarized in Table 1.

**Table 1:** Regression equations and p-values of the mixed effects model

**Variable**	**Solutions for the fixed effects using the mixed effect model**						**p-value**
	**Intercept**	**cos FA**	**cos**^**2**^**FA**	**cos**^**3**^**FA**	**Flexion/ extension**	**Natural/ TKA**	**Natural/ TKA**
Quadriceps load	52.88	−7.51	+0.23	−0.0011	+118.91	+3.79	0.28
Tibial rotation	−1.00	+0.067	−0.0011	+4.75e-6	−0.38	+2.66	0.001
Peak pressure	0.89	−0.058	0.0018	−8.83e-6	+0.45	+2.66	0.004
Peak pressure (ridge)	0.99	+0.063	0.0021	−0.00001	+0.48	−0.61	0.0001
Peak pressure (medial)	−0.10	−0.015	+0.00089	−4.00e-6	+0.27	−1.24	0.064
Peak pressure (lateral)	0.42	−0.032	+0.0011	−5.09e-6	+0.30	−0.16	0.011
Contact area	285.99	−0.30	+0.0097	-0.00014	+7.36	−0.14	<0.0001
Proximalization	8.55	+0.31	−0.0058	+ 0.000019	+2.51	+3.58	<0.0001

## Results

**Figure 3:**
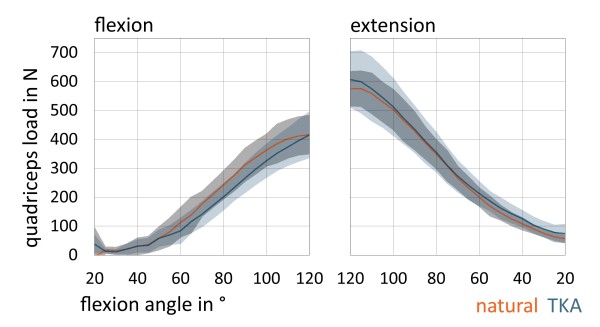
Charts are representing mean quadriceps load (in N) with standard deviation according to flexion and extension grade of the knee (between 20°-120°).

The load of quadriceps was comparable before and after TKA (p = 0.28). The mean minimum load in natural knee and after TKA was 15 N (±12 N) near extension (20° of flexion). The mean maximum load was 575 N (±60 N) in natural knee and after TKA 607 N (±96 N) during extension (near 120° of flexion) (Figure 3).

**Figure 4:**
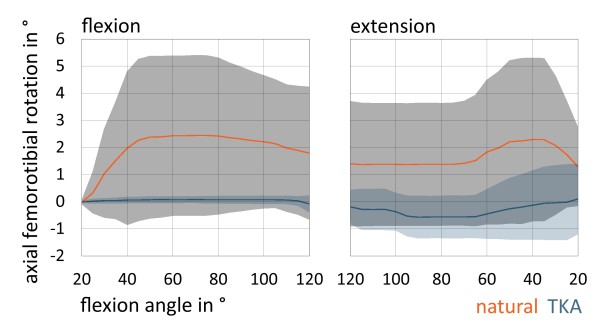
Charts are representing mean axial femorotibial rotation (in °; positive: internal rotation, negative: external rotation) with standard deviation in natural knee and after TKA.

All seven natural knees showed typical tibial internal rotation during flexion respectively external rotation with extension the so called screw-home rotation [26]. The maximum amount of axial rotation from 20° to 120° of knee flexion was 8.0°, the minimum amount was 0.1° (Figure 4). The average amount of axial rotation was 3.5°.

In contrast after TKA there was a significant lower axial tibial rotation with flexion and extension (p = 0.001). The maximum amount of axial rotation from 20° to 120° of knee flexion after TKA was 1.7°, the minimum amount was 0.6°. The average amount of axial rotation was 1.3°. Five knees showed internal rotation with flexion after TKA and two knees showed slight external rotation with flexion (average amount of reverse axial rotation 1.6°). One knee rotated internally until 100° and then rotated slightly externally until 120° of flexion.

There was a significant difference of mean retropatellar pressure between natural knee and after TKA. In the natural knee there was a mean peak pressure of 4.05 (+/−1.23) MPa and after TKA of 6.19 (+/−1.27) MPa (p = 0.004). After TKA peak pressure on the entire retropatellar surface was measured in 120° flexion of the knee. Natural knee in contrast had a peak pressure between 75° to 100° of flexion. Peak pressure decreased between 100° and 120° constantly and increased again with following extension in natural knee.

**Figure 5:**
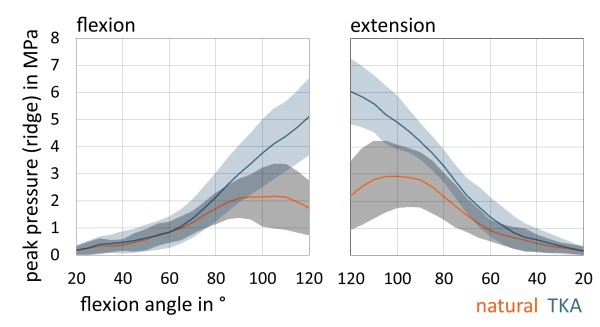
Charts are representing retropatellar mean peak pressure (in MPa) with standard deviation on the ridge of the patella according to flexion and extension grade of the knee (between 20°-120°) in natural knee and after TKA

Considering the subdivision of the retropatellar surface in ridge, medial and lateral surface we obtained significant differences on the ridge of the patella during complete flexion and extension cycle. After TKA there was a mean peak pressure of 6.09 (+/−1.31) MPa in contrast to natural knee with a peak pressure of 2.92 (+/−1.15) MPa (p < 0.0001) (Figure 5). On the medial retropatellar surface there was a mean peak pressure of 3.68 (+/−1.30) MPa preoperatively and 3.97 (+/−1.40) MPa postoperatively (p = 0.064). On the lateral retropatellar surface there was a mean peak pressure of 3.21 (+/−1.57) MPa in the natural knee and after TKA of 3.82 (+/−1.99) MPa (p = 0.011).

**Figure 6:**
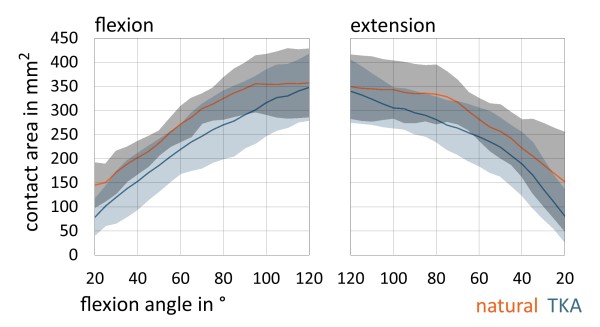
**Charts are representing mean retropatellar contact area (in mm**^**2**^**) with standard deviation on the entire retropatellar surface according to flexion and extension grade of the knee (between 20°-120°) in natural knee and after TKA.**

The maximum contact area was observed at deep flexion (Figure 6). Between 90° and 120° of flexion the mean contact area of the ridge was constantly lower in the natural knee compared to the findings after implantation of the prosthesis. Considering complete flexion and extension cycle medial and lateral retropatellar contact area was higher in natural knee than after TKA (p < 0.0001).

**Figure 7:**
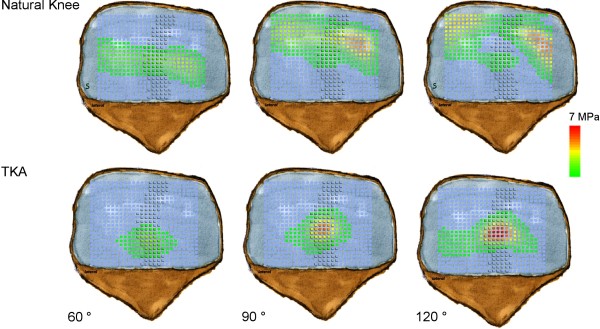
**Retropatellar pressure distribution in natural knee and after TKA in 60°, 90° and 120° of knee flexion.** (ridge = shaded area).

There was an apparent and significant proximalization of patella contact area on retropatellar surface during flexion for both trials (p < 0.0001, Figure 7). In natural knee we observed a proximalization of 21.42 mm (± 3.9 mm) and after TKA of 17.93 mm (± 2.2 mm).

## Discussion

Anterior knee pain is still an important complication and is causing a high percentage of unsatisfied patients after TKA [27]. The assumption that resurfacing the patella could prevent the phenomenon of anterior knee pain could not be proved [28-30]. The prospective, randomized clinical trial by Burnett et al. showed no difference between resurfacing and nonresurfacing after a 10 year follow-up in bilateral TKA. Deductive of their results Barrack et al. concluded that femoral component design might affect postoperative outcome concerning anterior knee pain more than the question to resurface or not [7]. Kulkarni et al. stated in their clinical study that design of the prosthetic trochlea might be most important for a good clinical outcome after TKA [9]. Varadarajan et al. found a significant difference between the physiologic and prosthetic trochlear anatomy, which could be only restored in parts by external rotation of the femoral component [10]. To further highlight this debate our study was conducted to analyze the effect on patellofemoral contact patterns after TKA.

In 1997, Matsuda et al. published their study concerning the effects of TKA on patellofemoral contact area and contact stress [31]. It is noteworthy that they only performed static measurements in six different flexion angles in five specimens. Fuchs et al. analyzed retropatellar pressure distribution in four different knee positions between 45°and 120° [13]. The joint contact area was subdivided into 9 areas and showed an increasingly load in the central areas of the retropatellar surface, however without identifying the anatomical ridge of the patella. This analysis was only performed during a loading of 5 seconds in four different knee positions with a constant quadriceps load of 280 N and only with a pressure sensitive film. Additionally the studies were performed with embalmed Thiel fixated specimens. The latest study presented to that subject showed also higher pressures in the central areas after TKA, although this study only was performed from 15° until 90 degree of knee flexion without subdividing the retropatellar surface [32]. In our study we analyzed retropatellar contact patterns before and after implantation of “standard” knee prosthesis concerning especially the ridge, medial and the lateral retropatellar surface from 20° to 120° of flexion. Especially on the ridge there was a highly significant increase of the retropatellar pressure of about 109% in maximum flexion after TKA compared to natural knee. This increasing patellofemoral contact forces might lead to anterior knee pain in vivo [9,11-13]. Interesting is that with higher flexion grades the central retropatellar pressure increases in TKA whereas in natural knee central retropatellar pressure is constantly decreasing (Figure 7). This is probably mainly due to the notch of the knee, which is guaranteeing a balanced load on the medial and lateral retropatellar surface and a descent of the ridge in the notch during flexion cycle. Surgeons should be aware that conformity of the trochlea and femoral condyles of the femoral component may not replicate normal anatomy of the knee. Thus conclusions might be drawn in terms of trochlea design of the femoral component or shaping the nonresurfaced patella. However, it has to be admitted, that other implant designs have not been tested in this setup.

Another aspect after TKA is the alternated knee kinematics compared to normal knee [33,34]. A multicenter fluoroscopy analysis of 1,027 knees showed especially in ACL-sacrificing TKAs a significant changing of axial femorotibial rotation patterns [16] in deep knee bending maneuver. Their study revealed a significant less overall rotation after TKA compared to natural knees independent of prosthesis design (mobile vs. fix bearing). Several other studies also showed a lower femorotibial rotation after TKA and in some cases even a reverse screw-home rotation [23,35-37]. This is similar to our results with a significant lower axial femorotibial rotation after TKA and two knees with reverse screw-home rotation. It is assumed that the lack of the anterior cruciate ligament and the failure to duplicate the geometry of the femoral and tibial condyles are causing different femorotibial rotation patterns after TKA compared to natural knee. Although femorotibial rotation is important for patellar tracking and to prevent patellofemoral shear forces [16]. With less femorotibial rotation after TKA patellofemoral problems might occur.

In addition flexion and extension moment are altering after TKA [17] and quadriceps strength is supposed to be an important factor for a good function of a TKA and to prevent anterior knee pain [18]. An alternated movement of the knee after TKA might lead to a higher quadriceps load and lead to earlier fatigue under physiologic strain such as squatting, rising from a chair or walking stairs. Indeed our results showed a slightly higher maximum quadriceps load of 5% after TKA, although this difference did not reach statistical significance. Ostermeier et al. showed in their study [19] an increase of 9% of quadriceps load for the posterior cruciate-retaining fixed-bearing group. Decreasing effectiveness of the lever arm as a consequence of the alternated knee kinematics is made responsible for this phenomenon. Hamstring muscles were although not simulated in this study, which could change the lever arm through more paradoxical anterior femoral translation [38-40] and might explain the higher percentage of quadriceps load.

In this study all specimens were tested with a ground reaction force of 50 N under a velocity of 3°/s from 20° to 120° flexion and back to 20° extension. Results of Müller et al. [21] and Victor et al. [22] showed that knee kinematic profiles differed between the nonloaded and loaded simulation in the knee rig. However both authors did not find relevant changes of the kinematic profiles with different loadings. Therefore an increased ground load may not improve a qualitative better outcome. Due to the ground force of 50 N the soft tissue of the specimens can be protected and same conditions of the specimens are more likely during the whole experiment. Furthermore the measured peak pressure of some specimens after TKA reached nearly the maximum capacity of the pressure sensor. An increased ground force would have resulted in a pressure sensor with an increased maximum capacity and consequential in a lower accuracy and sensitivity of this sensor. There is no standard in literature regarding the velocity of knee rigs. The test rig of Müller et al. performed the squat with 1°/s [21]. Another research group defined the velocity of the hip-joint according to the ankle-joint to 2.75 mm/s [41]. To combine an accurate force control with a realistic squat simulation a velocity of 3°/s was defined for this study (velocity of max. 10 mm/s; hip-joint in relation to ankle-joint).

Seven specimens were tested in this study which is a comparable number to other studies with similar questions [12,13,18,23,31]. However due to the number of specimens tested a type II error might not be fully excluded in our study. An important limitation of our study is that only one particular TKA design has been tested. Hence the results may not be transferred to all other implant designs. However, this is a world-wide known TKA-system of one major manufacturer on the market, which is currently used for total knee replacement [42] and our results may be applied for similar implants. Retropatellar contact patterns after resurfacing was not investigated as this was not the question of the study. As there was no patellofemoral arthritis the patellae would not have been resurfaced in vivo as well [43]. We also could not simulate daily activities like normal gait or walking stairs with our knee rig, but parts of a loading squat might be transmittable in parts of some of these activities. Finally we did not analyze modifications in implanting the femoral component e.g. external or internal rotation.

## Conclusions

In the presented setting we found a significant increase of pressure on the ridge of the retropatellar surface, which may be one important reason for anterior knee pain after TKA in vivo. In contrast mean quadriceps loading after implantation of knee prosthesis did not change significantly. The significant lower internal rotation with knee flexion after TKA might lead to a patella maltracking. Changing the design of the prosthesis or a special way of patella shaping might increase the conformity of the patella to trochlea to maintain natural patellofemoral contact patterns.

Further studies must be conducted to analyze the influence of different designs of prostheses on patellofemoral contact patterns, knee kinematics and quadriceps load after TKA.

## Abbreviations

ASCII: American standard code for information interchange; DOF: Degree of freedom; FA: flexion angle; MPa: Mega Pascal; TKA: Total knee arthroplasty.

## Competing interests

The authors declare no conflict of interest with the content of this study.
